# Thermoregulation of foraging honeybees on flowering plants: seasonal variability and influence of radiative heat gain

**DOI:** 10.1111/j.1365-2311.2011.01313.x

**Published:** 2011-10-20

**Authors:** Helmut Kovac, Anton Stabentheiner

**Affiliations:** Department of Zoology, Karl-Franzens-University of Graz, Universitätsplatz 2Graz, Austria

**Keywords:** Body temperature, flower, foraging, honeybee, season, thermoregulation

## Abstract

1. During nectar and pollen foraging in a temperate climate, honeybees are exposed to a broad range of ambient temperatures, challenging their thermoregulatory ability. The body temperature that the bees exhibit results from endothermic heat production, exogenous heat gain from solar radiation, and heat loss. In addition to profitability of foraging, season was suggested to have a considerable influence on thermoregulation. To assess the relative importance of these factors, the thermoregulatory behaviour of foragers on 33 flowering plants in dependence on season and environmental factors was investigated.

2. The bees (*Apis mellifera carnica* Pollman) were always endothermic. On average, the thorax surface temperature (*T*_th_) was regulated at a high and rather constant level over a broad range of ambient temperatures (*T*_th_ = 33.7–35.7°C, *T*_a_ = 10–27°C). However, at a certain *T*_a_, *T*_th_ showed a strong variation, depending on the plants from which the bees were foraging. At warmer conditions (*T*_a_ = 27–32°C) the *T*_th_ increased nearly linearly with *T*_a_ to a maximal average level of 42.6 °C. The thorax temperature excess decreased strongly with increasing *T*_a_ (*T*_th_−*T*_a_ = 21.6 − 3.6°C).

3. The bees used the heat gain from solar radiation to elevate the temperature excess of thorax, head, and abdomen. Seasonal dependance was reflected in a 2.7 °C higher mean *T*_th_ in the spring than in the summer. An anova revealed that season had the greatest effect on *T*_th_, followed by *T*_a_ and radiation.

4. It was presumed the foragers' motivational status to be the main factor responsible for the variation of *T*_th_ between seasons and different plants.

## Introduction

Honeybees need nectar and pollen to provide for their young bees and brood. Honey supplies energy for heat production to achieve a constant brood temperature and for overwintering in a temperate climate (Stabentheiner *et al*., 2003a, 2010). During foraging, bees are mostly highly endothermic. They may exhibit thoracic temperatures higher than 40 °C (e.g. Heinrich, 1979a; Cooper *et al*., 1985; Schmaranzer & Stabentheiner, 1988; Kovac & Schmaranzer, 1996; Schmaranzer, 2000; Kovac *et al*., 2010). Thermoregulatory investigations of honeybees during foraging on natural sources in their environment are very scarce. Heinrich (1979a) measured thoracic (core) temperatures of *Apis mellifera mellifera* and *Apis mellifera adansonii* Linnaeus during foraging on *Eucalyptus* sp., *Bidens pilosa* L., and *Petrea volubilis* L. Thoracic temperatures were regulated between 31 and 32 °C, differing insignificantly between the European honeybee and the African variety. Kovac and Schmaranzer (1996) measured body surface temperatures of honeybees (*Apis mellifera carnica* Pollman) foraging in the shade in the spring and summer at several different plants. The average thorax temperature varied in a broad range (*T*_th_ = 29.3–35.7°C, mean values per flower).

The body temperature of foraging insects is influenced by several environmental factors such as ambient air temperature, solar radiation (Willmer & Unwin, 1981), and convection (for an overview see Heinrich, 1993). The energy gain from solar radiation is of importance for the thermoregulation of foraging bees. An increase of the thorax temperature with increasing insolation was reported in Western honeybees arriving at the nest entrance after their foraging flights (Cena & Clark, 1972; Heinrich, 1979a; Cooper *et al*., 1985) and during nectar foraging (Heinrich, 1979a). Underwood (1991) reported the same for Indian honeybees collecting sugar syrup under sunny and overcast skies. Kovac *et al*. (2009a) investigated the influence of solar radiation on the thermoregulation of water-foraging wasps in detail. *Vespula* and *Polistes* increased the thorax temperature and reduced the active heat production as solar heat gain increased. In water-foraging honeybees, the relative contribution of endothermic heat production and heat gain from solar radiation on body temperature was observed by Kovac *et al*. (2010). Up to an ambient temperature of ∼30 °C, bees used solar heat gain for a dual purpose: to reduce energetic expenditure and to increase the thorax temperature by about 1–3 °C, in order to improve force production of flight muscles (Coelho, 1991a) and to speed up suction velocity (Kovac *et al*., 2010). The aim of the present study was to investigate the contribution of radiative heat gain on the bees' thermoregulation during foraging for nectar and pollen under natural conditions.

Kovac and Schmaranzer (1996), demonstrated in a comparison of honeybees foraging from 13 flowers, considerable variation of the thorax temperature. As a rule, the energy expenditure of individual foragers is balanced with the net energetic gains to the colony (Schmid-Hempel *et al*., 1985; Seeley *et al*., 1991; Seeley, 1995). The bees minimise the thermoregulatory costs during foraging by adapting their thorax temperature in response to the profitability of foraging at a food source and the colony's need for nectar and pollen (Stabentheiner & Schmaranzer, 1986, 1987; Dyer & Seeley, 1987; Schmaranzer & Stabentheiner, 1988; Waddington, 1990; Stabentheiner & Hagmüller, 1991; Underwood, 1991; Stabentheiner *et al*., 1995; Stabentheiner, 2001; Nieh *et al*., 2006; Sadler & Nieh, 2011). From these investigations, we know the thoracic temperature to vary in a broad range of ∼30–44 °C. As flowers differ considerably in their profitability, i.e. as they vary in the amount of pollen and concentration and flow of nectar, the distance between single blossoms, and because the bees adapt their thorax temperature to profitability, the bees' thorax temperature at a certain flower is not predictable from measurements at other flowers. Therefore, to get a broader overview of the foragers' thermoregulation in their temperate living space, we investigated them on flowers at different locations and environmental conditions.

Under Central European climate conditions, honeybee colonies undergo a typical seasonal population development, influenced by environmental and genetic parameters. The climax of the population strength and brood nest dimension is reached from the middle to the end of June (e.g. Seeley, 1985; Wille, 1985; Winston, 1987; Liebig, 1994; Imdorf *et al*., 1996). In spring, when the colonies have much brood and low food reserves, the bees should be more motivated to forage. In foraging honeybees, thorax temperature correlates with the insects' motivational state (e.g. Dyer & Seeley, 1987; Stabentheiner & Schmaranzer, 1987; Schmaranzer & Stabentheiner, 1988; Stabentheiner & Hagmüller, 1991; Underwood, 1991; Stabentheiner *et al*., 1995; Stabentheiner, 2001; Sadler & Nieh, 2011). Kovac and Schmaranzer (1996) presumed that season, beside ambient temperature, has an influence on thermoregulation. However, to test this hypothesis, data from more than 2 years and from multiple flowers were necessary, and measurements in sunshine had to be included (Kovac & Schmaranzer, 1996, had measured in shade). Our investigation covers a complete foraging season under Central European climate conditions. This allowed measurements over the entire range of ambient temperatures and solar radiation to which bees are probably exposed to during their foraging trips. Results should enable assessment of the relative importance of season and environmental factors.

## Materials and methods

### Animals, field site, and measuring conditions

Measuring locations were the botanical garden in Graz and several orchards and meadows near Graz, Austria, Central Europe. We investigated honeybees (*A. mellifera carnica*) foraging nectar and pollen on 33 different blossoms of flowers, shrubs and trees, and collecting water from a rainwater barrel. To cover the entire foraging season and range of ambient temperatures honeybees are exposed to under Central European climate conditions, measurements were made on 26 days from March to October in 2006 (Table 1). Measurements were performed in different weather conditions, from overcast sky to bright sunshine. If no flowers were available in shade, a patch of flowers was shaded by a sunshade.

**Table 1:** Summary statistics for the surface temperature of the head, thorax and abdomen of foraging honeybees on different flowering plants, and blossom surface temperature near the bees' mouthparts (*T*_blossom_), ambient temperature (*T*_a_), relative humidity (rel. hum.), and solar radiation (sol. rad.) for each single measuring day divided in three classes of solar radiation.

No.	Date	Plant	Rad. classes (W m^−2^)	*N*_bees_	*N*_means_	*T*_head_ (°C)	*T*_thorax_ (°C)	*T*_abdomen_ (°C)	*T*_blossom_ (°C)	*T*_a_ (°C)	Rel. hum. (%)	Sol. rad. (W m^−2^)
1	30 March 2006	*Crocus vernus*	<200	1	1	29.1 ± 0.0	37.7 ± 0.0	19.4 ± 0.0	15.3 ± 0.0	17.3	33.8	193.0
			200–500	16	250	29.4 ± 1.6	37.4 ± 1.6	19.8 ± 1.2	17.9 ± 1.2	18.4	30.5	369.7
			>500	1	6	27.9 ± 0.8	36.8 ± 2.2	22.1 ± 0.6	18.4 ± 1.2	16.8	33.6	535.7
2	4 April 2006	*Salix caprea*	<200	14	137	24.2 ± 2.1	33.7 ± 2.9	14.8 ± 1.0	12.8 ± 1.0	12.7	41.0	179.7
			200–500	24	183	25.5 ± 2.9	34.3 ± 3.4	16.3 ± 1.8	14.1 ± 1.8	13.1	39.5	249.6
			>500	24	365	28.3 ± 3.3	35.5 ± 3.4	18.9 ± 2.2	16.6 ± 3.2	14.1	38.2	903.8
3	20 April 2006	*Prunus armeniaca*	<200	7	112	30.6 ± 1.6	36.6 ± 1.7	20.9 ± 1.0	18.1 ± 1.3	18.2	40.7	176.7
			200–500	11	166	31.3 ± 1.9	36.4 ± 1.9	22.1 ± 1.8	18.6 ± 1.0	18.6	40.6	301.5
			>500	8	115	32.9 ± 2.1	36.4 ± 2.1	24.3 ± 1.6	19.5 ± 2.0	19.7	37.3	886.8
4	20 April 2006	*Cardamine pratensis*	<200	14	59	28.8 ± 1.9	34.7 ± 2.9	21.3 ± 1.5	18.0 ± 1.4	18.4	35.9	186.1
			200–500	14	69	29.0 ± 2.0	35.3 ± 2.6	21.6 ± 1.3	18.2 ± 1.1	18.9	37.2	211.0
			>500	25	253	30.1 ± 2.5	33.8 ± 3.3	23.3 ± 1.3	19.0 ± 1.2	18.7	37.1	814.0
5	20 April 2006	Water	<200	79	200	29.0 ± 2.0	37.1 ± 2.3	24.8 ± 1.9	21.4 ± 1.5	18.0	45.4	151.1
			200–500	18	43	31.4 ± 1.6	37.6 ± 2.5	29.6 ± 1.0	25.2 ± 1.6	19.5	37.5	317.7
			>500	4	7	32.6 ± 0.6	38.6 ± 1.8	30.5 ± 0.5	25.8 ± 1.5	18.8	30.1	678.7
6	22 April 2006	*Prunus sp*.	<200	16	130	30.1 ± 2.1	35.5 ± 2.2	23.0 ± 1.0	20.0 ± 1.5	20.7	47.0	158.1
			200–500	9	28	29.7 ± 1.9	35.0 ± 1.8	22.5 ± 0.7	19.2 ± 1.8	19.9	45.4	286.1
			>500	28	155	31.2 ± 2.8	34.9 ± 3.6	24.4 ± 1.4	20.9 ± 1.5	20.9	47.6	812.6
7	24 April 2006	*Cerasus avium*	<200	11	97	28.6 ± 1.6	33.4 ± 2.0	21.9 ± 1.0	19.0 ± 1.3	19.4	47.8	141.3
			200–500	4	20	28.7 ± 1.3	33.1 ± 2.0	23.2 ± 1.3	20.3 ± 1.4	18.5	51.2	355.9
			>500	9	29	27.5 ± 2.4	31.6 ± 2.5	22.5 ± 0.8	17.5 ± 4.9	19.2	52.8	673.8
8	24 April 2006	*Taraxacum officinalis*	<200	13	92	32.9 ± 1.8	37.7 ± 1.5	28.3 ± 1.9	23.9 ± 2.2	22.7	42.8	178.1
			200–500	9	34	32.8 ± 2.0	37.6 ± 1.2	29.8 ± 1.3	25.4 ± 2.7	22.5	39.8	212.8
			>500	23	212	37.2 ± 2.0	39.9 ± 1.7	34.7 ± 2.5	31.4 ± 2.9	24.0	41.8	981.4
9	4 May 2006	*Malus domestica*	<200	—	—	—	—	—	—	—	—	—
			200–500	17	71	24.7 ± 1.5	34.0 ± 1.8	19.1 ± 1.4	15.5 ± 0.7	15.8	56.4	403.5
			>500	19	109	27.8 ± 2.0	35.8 ± 2.3	21.8 ± 1.6	17.3 ± 1.2	16.8	50.4	862.2
10	4 May 2006	*Taraxacum officinalis*	<200	9	127	24.9 ± 1.5	36.0 ± 1.8	19.8 ± 1.2	16.7 ± 1.3	16.0	57.2	153.5
			200–500	6	78	26.2 ± 1.5	36.9 ± 1.3	20.4 ± 1.5	17.8 ± 1.4	16.5	56.1	243.2
			>500	4	24	29.5 ± 1.1	38.0 ± 1.1	25.0 ± 1.7	22.7 ± 2.7	18.4	49.7	893.8
11	8 May 2006	*Mahonia aquifolium*	<200	12	166	25.5 ± 1.5	35.7 ± 1.9	18.3 ± 1.1	14.6 ± 1.3	14.9	58.9	105.3
			200–500	22	199	26.1 ± 1.9	34.9 ± 1.6	19.8 ± 2.1	16.2 ± 1.7	16.1	53.5	251.0
			>500	7	50	28.7 ± 1.9	35.6 ± 1.1	23.3 ± 2.6	19.1 ± 2.1	18.4	54.6	793.9
12	11 May 2006	*Taraxacum officinalis*	<200	15	211	27.2 ± 1.0	35.9 ± 1.4	21.5 ± 0.8	19.0 ± 1.2	17.6	53.3	71.1
			200–500	9	63	27.8 ± 2.0	35.0 ± 1.8	23.2 ± 3.6	20.2 ± 2.8	17.9	51.0	287.9
			>500	30	276	32.7 ± 2.3	36.3 ± 1.9	32.4 ± 2.9	27.1 ± 3.3	20.9	76.1	1105.0
13	11 May 2006	*Mahonia aquifolium*	<200	16	126	26.7 ± 1.4	34.3 ± 1.7	21.6 ± 1.4	18.1 ± 1.1	18.3	39.6	179.3
			200–500	11	97	26.5 ± 1.2	33.9 ± 1.4	21.7 ± 1.6	18.7 ± 1.5	18.5	39.2	225.9
			>500	30	259	30.1 ± 2.1	34.4 ± 1.6	25.3 ± 2.1	21.3 ± 2.6	19.1	40.4	883.2
14	18 May 2006	*Brassica napus*	<200	1	3	29.3 ± 0.9	37.8 ± 2.0	25.6 ± 1.3	21.4 ± 0.1	22.1	55.9	192.3
			200–500	42	139	29.7 ± 1.6	36.0 ± 1.8	25.3 ± 1.3	22.3 ± 1.2	22.7	55.1	303.0
			>500	38	198	30.6 ± 1.6	35.8 ± 1.8	26.5 ± 1.1	23.0 ± 1.0	22.9	55.1	818.9
15	23 May 2006	*Ranunculus bulbosus*	<200	9	75	29.2 ± 1.2	35.3 ± 1.3	26.5 ± 1.5	23.3 ± 1.4	22.3	64.2	128.0
			200–500	4	21	30.6 ± 1.0	36.8 ± 1.4	25.3 ± 1.8	22.5 ± 1.3	21.2	66.4	233.7
			>500	14	84	33.3 ± 1.4	37.2 ± 1.3	30.7 ± 1.3	26.7 ± 1.6	23.6	63.7	966.4
16	23 May 2006	*Crepis sp*.	<200	—	—	—	—	—	—	—	—	—
			200–500	15	94	28.3 ± 1.0	32.5 ± 1.4	25.6 ± 0.8	23.4 ± 1.0	22.1	61.8	404.1
			>500	9	58	30.0 ± 1.3	33.2 ± 1.1	27.6 ± 1.4	25.1 ± 1.2	23.2	61.9	721.5
17	8 June 2006	*Angelica archangelica*	<200	11	85	26.2 ± 1.3	33.0 ± 1.8	19.0 ± 1.0	15.0 ± 1.0	16.3	54.4	154.8
			200–500	26	146	26.3 ± 1.7	31.6 ± 1.4	19.8 ± 2.3	15.7 ± 2.0	16.5	56.4	302.7
			>500	30	142	29.9 ± 2.1	32.7 ± 1.5	25.6 ± 2.4	19.1 ± 1.6	18.4	50.6	1027.9
18	8 June 2006	Water	<200	12	122	25.6 ± 1.9	35.8 ± 2.8	23.7 ± 1.0	19.3 ± 0.7	16.6	57.4	155.6
			200–500	5	17	26.7 ± 1.2	35.7 ± 1.7	24.7 ± 1.8	20.4 ± 1.3	16.3	57.1	307.6
			>500	—	—	—	—	—	—	—	—	—
19	8 June 2006	*Rubus idaeus*	<200	8	80	24.3 ± 1.4	30.9 ± 1.6	18.9 ± 0.8	16.5 ± 0.8	17.2	50.3	157.3
			200–500	17	136	24.7 ± 1.2	31.1 ± 1.4	19.2 ± 1.0	16.8 ± 1.0	17.2	48.5	254.3
			>500	1	7	27.4 ± 0.6	32.5 ± 1.0	24.8 ± 1.3	20.9 ± 1.5	19.0	51.1	969.4
20	12 June 2006	*Cornus sanguinea*	<200	27	300	30.3 ± 1.2	35.5 ± 2.0	26.5 ± 0.9	22.6 ± 1.0	22.8	41.1	84.6
			200–500	1	1	35.2 ± 0.0	38.2 ± 0.0	32.2 ± 0.0	27.0 ± 0.0	26.3	36.2	465.0
			>500	34	297	33.2 ± 2.1	36.0 ± 2.0	30.9 ± 2.0	24.8 ± 2.2	23.8	41.7	854.2
21	20 June 2006	*Rubus sp*.	<200	37	212	32.7 ± 2.1	35.7 ± 2.3	31.6 ± 1.7	28.2 ± 1.5	28.2	42.5	98.7
			200–500	1	3	37.5 ± 0.8	40.2 ± 0.8	35.8 ± 0.1	32.3 ± 1.3	29.2	45.5	479.0
			>500	39	200	36.6 ± 1.4	39.5 ± 1.5	35.2 ± 2.0	31.4 ± 1.8	28.9	43.1	983.5
22	20 June 2006	*Aegopodium podagraria*	<200	20	227	32.7 ± 1.2	36.0 ± 2.1	30.2 ± 0.8	26.9 ± 0.7	29.6	73.1	101.5
			200–500	1	1	37.1 ± 0.0	38.1 ± 0.0	35.7 ± 0.0	27.6 ± 0.0	29.8	47.5	440.0
			>500	25	285	37.6 ± 2.1	40.1 ± 1.9	35.3 ± 2.1	28.6 ± 0.9	30.5	54.8	1048.2
23	27 June 2006	*Castanea sativa*	<200	12	89	32.7 ± 1.5	35.1 ± 1.7	30.2 ± 1.4	28.1 ± 0.9	28.3	57.1	145.2
			200–500	—	—	—	—	—	—	—	—	—
			>500	13	70	36.6 ± 2.0	38.5 ± 1.6	33.8 ± 1.8	30.8 ± 2.1	29.0	58.0	884.3
24	27 June 2006	*Trifolium repens*	<200	5	23	34.9 ± 0.9	38.4 ± 1.3	31.4 ± 1.0	29.5 ± 0.8	29.5	74.1	190.2
			200–500	9	118	35.2 ± 0.9	38.9 ± 1.2	31.6 ± 0.8	29.7 ± 0.7	29.4	72.6	244.4
			>500	11	150	40.0 ± 1.7	41.9 ± 1.4	35.9 ± 1.6	32.5 ± 1.4	31.5	66.2	999.6
25	30 June 2006	*Tilia cordata*	<200	—	—	—	—	—	—	—	—	—
			200–500	29	247	27.8 ± 1.9	30.8 ± 2.6	24.2 ± 1.3	21.7 ± 0.8	22.5	58.2	369.9
			>500	10	114	33.2 ± 2.7	34.8 ± 2.4	29.8 ± 2.5	25.6 ± 2.0	25.1	55.5	1031.5
26	6 July 2006	*Lavendula sp*.	<200	21	226	28.3 ± 2.0	32.2 ± 2.0	24.5 ± 1.8	23.5 ± 1.7	24.9	51.4	80.1
			200–500	—	—	—	—	—	—	—	—	—
			>500	26	194	31.1 ± 2.1	33.4 ± 1.9	27.8 ± 2.1	25.8 ± 2.0	25.6	52.0	926.1
27	12 July 2006	*Cirsium arvense*	<200	14	218	29.3 ± 1.3	30.8 ± 1.7	28.4 ± 1.0	26.4 ± 0.6	27.3	60.5	122.1
			200–500	1	1	30.2 ± 0.0	32.2 ± 0.0	28.9 ± 0.0	27.1 ± 0.0	29.2	51.7	212.0
			>500	18	137	35.4 ± 1.6	36.8 ± 1.6	33.7 ± 1.8	31.8 ± 2.0	28.7	60.1	892.6
28	19 July 2006	*Begonia semperflorens*	<200	14	98	32.1 ± 3.4	34.6 ± 3.2	30.6 ± 3.4	27.2 ± 2.5	28.5	40.9	115.9
			200–500	11	39	35.5 ± 3.5	37.7 ± 3.4	33.8 ± 3.1	30.1 ± 2.2	30.5	43.0	256.4
			>500	20	104	37.2 ± 2.9	39.1 ± 2.5	35.6 ± 2.9	32.0 ± 2.3	29.9	41.9	1027.0
29	19 July 2006	*Mentha longifolia*	<200	9	104	30.6 ± 2.8	32.2 ± 3.1	30.0 ± 2.5	27.4 ± 2.1	27.8	40.8	109.3
			200–500	—	—	—	—	—	—	—	—	—
			>500	12	110	37.8 ± 0.9	39.5 ± 0.8	36.2 ± 1.0	31.9 ± 1.4	29.5	40.9	1073.1
30	23 August 2006	*Cirsium oleraceum*	<200	—	—	—	—	—	—	—	—	—
			200–500	15	75	29.7 ± 1.5	37.4 ± 2.9	26.9 ± 1.2	24.1 ± 1.3	21.4	59.4	323.4
			>500	4	13	31.0 ± 1.3	34.7 ± 1.9	28.1 ± 1.5	24.5 ± 1.2	23.0	55.4	1075.8
31	23 August 2006	Water	<200	—	—	—	—	—	—	—	—	—
			200–500	15	75	29.7 ± 1.5	37.4 ± 2.9	26.9 ± 1.2	24.1 ± 1.3	21.4	59.4	323.4
			>500	4	13	31.0 ± 1.3	34.7 ± 1.9	28.1 ± 1.5	24.5 ± 1.2	23.0	55.4	1075.8
32	28 August 2006	*Helianthus annuus*	<200	9	130	27.4 ± 0.8	29.8 ± 1.6	24.7 ± 0.7	25.8 ± 1.2	22.0	47.4	119.4
			200–500	5	58	29.3 ± 0.8	30.0 ± 0.9	26.6 ± 1.1	29.6 ± 1.3	21.9	44.2	370.3
			>500	14	167	34.0 ± 2.5	34.9 ± 2.8	31.5 ± 2.9	34.1 ± 2.6	23.4	44.7	768.9
33	28 August 2006	*Zinnia sp*.	<200	10	98	26.2 ± 1.5	28.7 ± 1.6	24.9 ± 1.4	23.1 ± 1.2	21.9	46.9	97.6
			200–500	2	6	28.9 ± 0.8	31.2 ± 1.3	27.5 ± 1.8	25.2 ± 1.6	22.0	46.8	466.0
			>500	8	37	33.0 ± 2.3	34.8 ± 2.3	31.2 ± 2.5	30.4 ± 3.0	23.4	45.4	923.0
34	6 September 2006	*Solidago gigantea*	<200	—	—	—	—	—	—	—	—	—
			200–500	3	20	33.4 ± 1.0	35.1 ± 0.6	31.2 ± 1.3	28.1 ± 1.3	26.1	59.1	458.0
			>500	19	161	32.7 ± 1.5	34.5 ± 1.5	29.7 ± 1.6	26.5 ± 1.5	25.3	56.2	608.9
35	10 September 2006	*Solidago gigantea*	<200	14	172	27.1 ± 1.1	31.5 ± 1.6	22.5 ± 0.9	20.6 ± 0.8	21.2	50.4	73.4
			200–500	—	—	—	—	—	—	—	—	—
			>500	24	166	31.9 ± 1.9	33.8 ± 1.9	28.6 ± 2.2	24.4 ± 1.9	23.8	50.7	679.7
36	11 September 2006	*Sedum spectabile*	<200	25	132	27.9 ± 1.8	32.5 ± 2.4	24.5 ± 1.4	21.9 ± 1.1	21.6	44.5	108.9
			200–500	—	—	—	—	—	—	—	—	—
			>500	29	177	34.9 ± 1.9	37.6 ± 1.7	34.2 ± 2.7	28.8 ± 2.8	24.4	46.3	793.6
37	11 September 2006	*Echinacea purpurea*	<200	15	83	25.8 ± 1.6	29.0 ± 2.4	23.7 ± 1.7	23.5 ± 2.2	21.3	43.3	106.7
			200–500	—	—	—	—	—	—	—	—	—
			>500	18	111	30.5 ± 1.4	33.0 ± 1.2	28.5 ± 1.8	29.4 ± 2.4	22.2	46.0	817.6
38	2 October 2006	*Aster sp*.	<200	68	149	26.9 ± 1.0	31.0 ± 1.5	22.7 ± 1.1	20.7 ± 1.1	20.7	76.5	161.0
			200–500	105	211	28.5 ± 1.8	31.1 ± 1.7	25.7 ± 2.3	23.6 ± 2.0	22.5	69.9	327.3
			>500	23	40	30.3 ± 2.2	32.6 ± 1.8	28.0 ± 2.2	25.4 ± 2.2	23.6	68.6	553.7
39	9 October 2006	*Aster sp*.	<200	61	168	27.1 ± 1.6	33.4 ± 2.3	22.4 ± 1.0	19.6 ± 0.8	19.5	56.3	64.5
			200–500	—	—	—	—	—	—	—	—	—
			>500	81	224	30.1 ± 1.6	32.9 ± 1.5	26.8 ± 1.9	22.8 ± 2.0	20.3	56.5	593.6
40	23 October 2006	*Sinapis arvensis*	<200	6	21	26.8 ± 2.1	33.1 ± 3.4	21.4 ± 1.7	18.8 ± 1.0	19.9	63.9	100.5
			200–500	15	66	26.7 ± 1.5	33.4 ± 1.6	19.8 ± 1.3	17.2 ± 1.4	17.2	70.1	359.3
			>500	19	82	28.6 ± 1.7	33.8 ± 2.0	22.7 ± 1.6	19.5 ± 1.7	18.6	65.6	670.3
41	23 October 2006	*Helianthus annuus*	<200	11	103	27.1 ± 1.6	29.3 ± 1.7	25.2 ± 2.0	25.0 ± 2.6	20.8	62.0	108.8
			200–500	7	38	28.3 ± 2.9	30.6 ± 2.7	24.1 ± 3.2	27.4 ± 3.2	18.3	67.8	352.5
			>500	17	133	34.5 ± 2.8	35.7 ± 2.8	31.6 ± 2.9	33.3 ± 3.1	21.2	63.2	638.6

Data presented as means ± SD or only means. *N*_bees_ = number of measured bees, *N*_means_ = number of measurements.

### Measurements

The bees were filmed during the foraging stays at the blossoms (if possible from landing until takeoff) with an infrared camera (ThermaCam SC2000 NTS, FLIR, Stockholm, Sweden). We used infrared thermography because it allows temperature measurements without contact and behavioural impairment (e.g. Stabentheiner & Schmaranzer, 1987; Schmaranzer & Stabentheiner, 1988; Kleinhenz *et al*., 2003; Kovac *et al*., 2009a,b; Stabentheiner *et al*., 2010). In addition, it allows simultaneous temperature monitoring of all body parts during the entire foraging stay at one blossom. This is especially important in insects with a variable body temperature like honeybees. The behaviour of the insects was not impaired, which would not have been possible with ‘grab and stab’ methods with thermocouples or thermoneedles (Stone & Willmer, 1989). This outweighs the disadvantage of the method, which measures surface and not core temperatures. The surface temperature of a thorax heated to 40 °C at an ambient temperature of 21.5 °C is ∼1 °C below the subcuticular temperature (Stabentheiner & Schmaranzer, 1987; B. Heinrich, pers. comm.). The infrared camera was calibrated periodically by slotting in a self-constructed peltier-driven reference source of known temperature and emissivity (for details of calibration see Stabentheiner & Schmaranzer, 1987; Schmaranzer & Stabentheiner, 1988). Thermographic data were stored digitally with a 14-bit resolution on a portable computer (DOLCH Flexpac-400-XG, Munich, Germany) at a rate of 3–5 frames s^−1^. On 3 days, in addition to the nectar-gathering bees, water-collecting bees foraging at a rainwater barrel a few metres away from the nectar-foragers were also measured.

The ambient air temperature (*T*_a_) was measured near the foraging bees (∼1–5 cm) with thermocouples. In the near vicinity of the insects (<1 m), we also measured the relative humidity with NTC-sensors (in shade) and the solar radiation with a miniature global radiation sensor (FLA613-GS mini spezial, Ahlborn, Holzkirchen, Germany). Care was taken so that the radiation sensor was exposed to the same ambient conditions as the foraging bees. The temperature and radiation data were stored every 2 s with ALMEMO data loggers (Ahlborn, Holzkirchen, Germany).

### Data evaluation and statistics

**Fig. 1:**
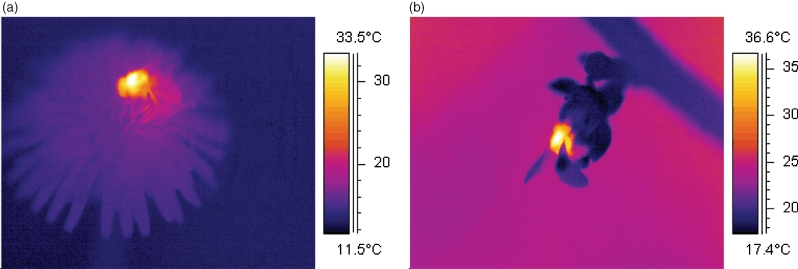
Thermograms of foraging honeybees on dandelion (*Taraxacum officinalis*, a) and apricot (*Prunus armeniaca*, b). (a) *T*_thorax_ = 34.6, *T*_head_ = 26.0, *T*_abdomen_ = 21.7°C, *T*_blossom_ = 19.3°C, *T*_a_ = 17.3°C, radiation = 66 W m^−2^. (b) *T*_thorax_ = 39.8, *T*_head_ = 33.6, *T*_abdomen_ = 22.7°C, *T*_blossom_ = 21.0°C, *T*_a_ = 18.7°C, radiation = 199 W m^−2^.

**Fig. 2:**
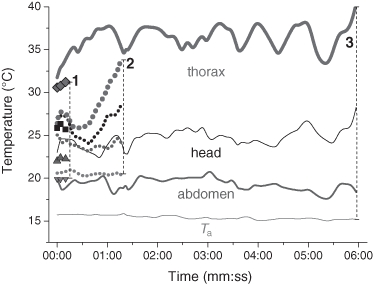
Body and ambient temperatures (*T*_a_) of a bee's short foraging stay on *Aster sp*. (1, symbols), a stay of medium duration on *Cirsium oleraceum* (2, dotted lines) and a long lasting stay on *Taraxacum officinalis* (3, continous lines). From the top to the bottom: thorax (red), head (black), abdomen (blue), and T_a_ (green).

The temperature of the three bee body parts and of the blossoms' surfaces (in close vicinity to the bees' mouthparts) was calculated from the infrared thermograms (Fig. 1) by means of the AGEMA Research software (FLIR, Stockholm, Sweden) controlled by a self-written Excel VBA-macro (Microsoft Corporation, Santa Rosa, California). The environmental data were automatically extracted from the datalogger files. Values of the body temperature during foraging were taken in regular intervals of about 3–5 s immediately after the insects' landing until their takeoff. This interval was chosen, because bees are able to increase or decrease body temperature within this time and temperature could vary considerably during foraging on one blossom (Fig. 2). The surface temperatures of the head (*T*_hd_), thorax (*T*_th_) and abdomen (*T*_ab_) were calculated with an infrared emissivity of 0.97, determined for the honeybee cuticle (Stabentheiner & Schmaranzer, 1987; Schmaranzer & Stabentheiner, 1988). Because the ThermaCam works in the long-wave infrared range (7.5–13 µm), the reflected radiation from the bees' cuticle produced only a small measurement error (0.2 °C for 1000 W m^−2^), which was compensated for. In this way we reached an accuracy of 0.7 °C for the body surface temperature of the bees at a sensitivity of <0.1 °C. The blossom surface temperature was calculated with an infrared emissivity of 0.95, representing a typical value for plants (Lamprecht *et al*., 2006).

The temperature gradient between the thorax and the ambient air (thorax temperature excess = *T*_thorax_−*T*_a_) was used as a measure to assess the bees' endothermic capability. To evaluate the influence of the radiative heat gain on the body temperature, three classes of solar radiation were established: shade, <200 W m^−2^, overcast sky, 200–500 W m^−2^, and sunshine, >500 W m^−2^. The mean of all foraging bees on one blossom type was calculated and values were divided into the three radiation classes. The values for the temperature excess of the head and abdomen were calculated in the same way.

The relationship between body temperature, temperature excess, and *T*_a_ was described by linear, exponential or polynomial regression functions and tested with anova. Data analysis and statistics were performed using the Statgraphics package (Statistical Graphics Corporation, Warrenton, Virginia) and ORIGIN software (OriginLab Corporation, Northampton, Massachusetts).

## Results

In 2006, we measured honeybees (*A. mellifera carnica*) foraging on 33 different flowering plants. Figure 1 shows thermograms of foraging bees on dandelion and apricot blossoms. From 1666 single forging stays we got 12 685 thermograms and evaluated the body surface temperatures of the head (*T*_hd_), thorax (*T*_th_), and abdomen (*T*_ab_) as well as the blossom surface temperature (*T*_blossom_) where the bees were sucking. We covered the complete foraging season (March–October) and the entire range of ambient temperatures (*T*_a_ =∼ 10–33°C) and solar radiation (50–1400 W m^−2^) to which they are likely to be exposed in their natural environment during a foraging trip in Central Europe. It must be noted that the investigated flowers often deliver both nectar and pollen (see Droege, 1989). We were unable to determine the relation of nectar and pollen load in the free-ranging individuals.

### Body temperature and blossom surface temperature

**Fig. 3:**
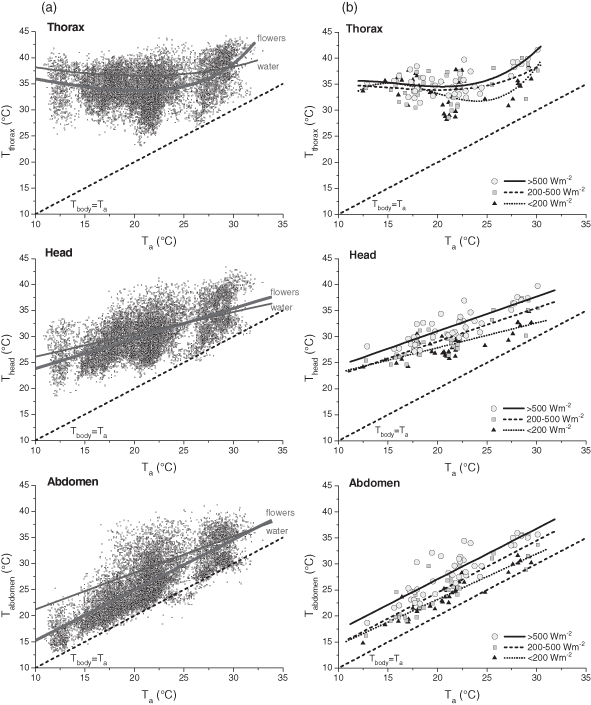
Surface temperature of the thorax, head and abdomen of foraging honeybees in dependence on ambient temperature (*T*_a_). (a) Foraging on flowering plants (dots are single values; bold red lines are regressions), and foraging water (thin blue lines; from Kovac *et al*., 2010). (b) Means per flowering plant and day at three different classes of solar radiation. Equations for linear and non-linear regressions, number of observations, and regression statistics in Table 2.

The body surface temperatures during nectar and pollen collection on one blossom were not constant but fluctuated, especially during longer-lasting stays (Fig. 2). The continuous measurement with infrared thermography enabled the registration of this variability within the foraging stay. The mean body surface temperatures per plant and date varied in a wide range, *T*_th_ from 23.2 to 44.2 °C, *T*_hd_ from 18.6 to 43.2 °C, and *T*_ab_ from 13.0 to 41.3 °C at ambient temperatures from 10.8 to 32.9 °C. A plot of all measurement data (Fig. 3) shows that at ambient temperatures of about 10–27 °C, *T*_th_ was regulated rather independent of *T*_a_ on average. At *T*_a_ > ∼27°C, however, it increased nearly linearly with *T*_a_ (Fig. 3). The head and abdomen exhibited a stronger dependence on *T*_a_ but both of them were regulated well above *T*_a_. The head was warmer and better regulated than the abdomen (Fig. 3). The abdominal temperature increased nearly linearly with *T*_a_. The relation of body temperature and ambient air temperature could be described best with an exponential function for the thorax (radiation: 0–1400 W m^−2^, *R*^2^ = 0.16185, Fig. 3, Table 2; A – E are the fit parameters):


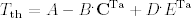
(1)

and with a simple linear regression for the head and the abdomen (radiation: 0–1400 W m^−2^, head: *R*^2^ = 0.41795, abdomen: *R*^2^ = 0.64091, Fig. 3, Table 2):


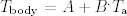
(2)

**Table 2:** Equations of linear and non-linear regressions for the temperature of the thorax (*T*_th_), head (*T*_hd_), and abdomen (*T*_ab_) of honeybees foraging on flowers or foraging for water (^*^, Kovac *et al*., 2010), and of the blossom temperature, in dependence on ambient temperature (*T*_a_) and solar radiation (Fig. 3).

Body part	Radiation (W m^−2^)	Equations	*R*^2^	*P*	*N*
		Bees on flowers			
Thorax	0–1400	*T*_th_ = 10.15733 + 29.83926 × 0.98433*T*_a_ + 0.05332 × 1.19123*T*_a_	0.16185	—	12685
	<200	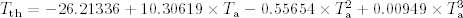	0.20651	—	38
	—	*T*_th_ = 33.67525 + 0.00041 ×*T*_a_	0.00000	>0.05	38
	200–500	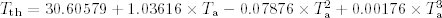	0.18030	—	34
	—	*T*_th_ = 30.22421 + 0.21173 ×*T*_a_	0.10899	>0.05	34
	>500	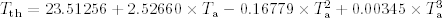	0.48709	—	40
	—	*T*_th_ = 28.90354 + 0.30973 ×*T*_a_	0.26565	<0.001	40
Head	0–1400	*T*_hd_ = 18.00959 + 0.58012 ×*T*_a_	0.41795	<0.0001	12503
	<200	*T*_hd_ = 18.32716 + 0.47753 ×*T*_a_	0.59942	<0.0001	38
	200–500	*T*_hd_ = 16.13789 + 0.64590 ×*T*_a_	0.72383	<0.0001	34
	>500	*T*_hd_ = 17.69125 + 0.66659 ×*T*_a_	0.72718	<0.0001	40
Abdomen	0–1400	*T*_ab_ = 5.56068 + 0.96864 ×*T*_a_	0.64091	<0.0001	12645
	<200	*T*_ab_ = 5.71561 + 0.87379 ×*T*_a_	0.87821	<0.0001	38
	200–500	*T*_ab_ = 4.54687 + 0.99761 ×*T*_a_	0.82406	<0.0001	34
	>500	*T*_ab_ = 7.57345 + 0.97364 ×*T*_a_	0.76073	<0.0001	40
		Bees foraging water^*^			
Thorax	0–1200	*T*_th_ = −10.25678 + 1.68281 × 1.06859*T*_a_ + 50.40771 × 0.98906*T*_a_	0.18480	—	11340
Head	0–1200	*T*_hd_ = 21.86636 + 0.42776 ×*T*_a_	0.59319	<0.0001	11290
Abdomen	0–1200	*T*_ab_ = 14.22541 + 0.70079 ×*T*_a_	0.81478	<0.0001	11334
		Blossom surface temperature			
	0–1200	*T*_bl_ = 3.39642 + 0.94273 ×*T*_a_	0.60852	<0.0001	11340
	<200	*T*_bl_ = 2.19283 + 0.91810 ×*T*_a_	0.87219	<0.0001	38
	200–500	*T*_bl_ = 3.30773 + 0.92522 ×*T*_a_	0.73901	<0.0001	34
	>500	*T*_bl_ = 4.05296 + 0.98745 ×*T*_a_	0.64128	<0.0001	40

*R*^2^ = squared correlation coefficient, *P* = probability, *N* = means per flower and day (<50) or number of measurements.

At a low *T*_a_ of 10 °C, the average values of *T*_th_, *T*_hd_, and *T*_ab_ derived from the regression lines were 35.6, 24.3, and 16.0 °C, respectively. In the medium range of *T*_a_, at about 20 °C, the *T*_th_ decreased to 33.7 °C, the *T*_hd_ increased to 29.6, and the *T*_ab_ increased to 24.9 °C. At the highest *T*_a_ measured (∼33 °C), *T*_th_, *T*_hd_, and *T*_ab_ increased to 44.4, 37.2, and 37.5 °C, respectively. In order to allow a comparison of the results of flower-visiting bees with water-foraging honeybees (from the paper of Kovac *et al*., 2010), the regression lines for the three body parts of the water foraging bees are also displayed in Fig. 3 (for statistical details see Table 2).

Plotting the body temperature in dependence on three levels of solar radiation (<200, 200–500, >500 W m^−2^; Fig. 3) revealed that bees foraging in sunshine were mostly warmer than bees foraging in shade. The relation of thorax temperature and ambient air temperature could be described best with a polynomial function (radiation: <200 W m^−2^: *R*^2^ = 0.20651, 200–500 W m^−2^: *R*^2^ = 0.18030, >500 W m^−2^: *R*^2^ = 0.48709, Fig. 3, Table 2; A – D are the fit parameters):


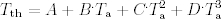
(3)

and with a simple linear regression [eqn (2)] for the head (radiation <200 W m^−2^: *R*^2^ = 0.59942, 200–500 W m^−2^: *R*^2^ = 0.72383, >500 W m^−2^: *R*^2^ = 0.72718) and the abdomen (radiation <200 W m^−2^: *R*^2^ = 0.87821, 200–500 W m^−2^: *R*^2^ = 0.82406, >500 W m^−2^: *R*^2^ = 0.76073). For further statistical and graphical details see Table 2 and Fig. 3. The temperature difference between >500 and <200 W m^−2^ as estimated from the regression lines of Fig. 3 was smaller at low and greater at high *T*_a_ (*T*_a_ = 12°C: difference *T*_th_ = 2.0, *T*_hd_ = 1.7, *T*_ab_ = 3.0°C; *T*_a_ = 30°C: difference *T*_th_ = 3.3, *T*_hd_ = 5.0, *T*_ab_ = 4.8°C).

**Fig. 4:**
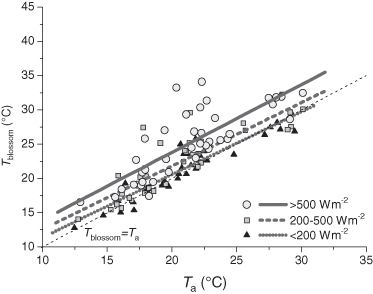
Temperature of the blossom surface near the honeybee mouthparts (means per flowering plant and day) in dependence on ambient temperature (*T*_a_) at three different classes of solar radiation. Equations of linear regressions, number of observations, and regression statistics in Table 2.

The blossom surface temperature (range *T*_bl_ = 9.5–42.2°C) measured closely beside the bees' mouthparts increased linearly in dependence on *T*_a_ at all three categories of radiation (Fig. 4, Table 1, statistical details in Table 2). In sunshine the blossoms' temperature was about 4 °C elevated above the ambient air temperature. Under (partly) overcast skies (200–500 W m^−2^) the *T*_bl_ was also always higher than the ambient air temperature. However, the blossoms' temperature in shade was similar to the ambient air. The three regression lines differed significantly (anova, *P* < 0.0001, *F*-Ratio = 68.35, d.f. = 5), and the intercepts of values in sunshine versus the two other categories of radiation were also significantly different (*P* < 0.01; *F*-Ratio = 6.83, 9.53, 36.32; d.f. = 1).

### Temperature excess and solar radiation

The bees were always endothermic as the thorax (the centre of heat production) was clearly more elevated above the ambient air than were the other body parts. The thorax temperature excess (*T*_th_−*T*_a_) depended strongly on *T*_a_. It decreased significantly with *T*_a_ in the sunshine and in the shade (values calculated from linear regressions in Table 3; *T*_th_−*T*_a_ = 20.6 − 8.2°C at *T*_a_ = 12–30°C and radiation >500 W m^−2^; *T*_th_−*T*_a_ = 21.6 − 3.6°C at *T*_a_ = 12–30°C and radiation <200 W m^−2^; *P* < 0.0001). The temperature excess of the intermediate radiation range (overcast sky, 200–500 W m^−2^) showed a similar course. An anova confirmed the difference in thorax temperature excess between sunshine and shade (*P* < 0.01, *F*-ratio = 11.01, d.f. = 1 for intercepts, and *P* < 0.05, *F*-ratio = 4.61, d.f. = 1 for slopes).

**3:** Equations of linear regressions for the temperature excess (*T*_body_−*T*_a_) of honeybees foraging on flowers in dependence on ambient temperature (*T*_a_), for three classes of solar radiation.

Body part	Radiation (W m^−2^)	Equations	*R*^2^	*P*	*N*
Thorax	<200	*T*_th_ = 33.67525 − 0.99959 ×*T*_a_	0.68966	<0.0001	38
	200–500	*T*_th_ = 30.22421 − 0.78827 ×*T*_a_	0.62899	<0.0001	34
	>500	*T*_th_ = 28.90354 − 0.69027 ×*T*_a_	0.64243	<0.0001	40
Head	<200	*T*_hd_ = 18.32716 − 0.52247 ×*T*_a_	0.64175	<0.0001	38
	200–500	*T*_hd_ = 16.13789 − 0.35410 ×*T*_a_	0.44063	<0.0001	34
	>500	*T*_hd_ = 17.69125 − 0.33341 ×*T*_a_	0.40007	<0.0001	40
Abdomen	<200	*T*_ab_ = 5.71561 − 0.12621 ×*T*_a_	0.13078	0.02569	38
	200–500	*T*_ab_ = 4.54687 − 0.00239 ×*T*_a_	0.00003	0.97678	34
	>500	*T*_ab_ = 7.57345 − 0.02636 ×*T*_a_	0.00232	0.76767	40

*R*^2^ = squared correlation coefficient, *P* = probability, *N* = number of means per flower and day.

The excess temperature of the head decreased with *T*_a_ as well, but the slopes were somewhat flatter than for the thorax. The decrease was still significant (*P* < 0.0001, Table 3). However, the temperature excess of the abdomen decreased with *T*_a_ only in the shade (*P* < 0.05) and remained constant between 12 and 33 °C in the sunshine and overcast sky (Table 3). An anova confirmed the difference in temperature excess between the sunshine and the shade (head: *P* < 0.0001, *F*-ratio = 75.98, d.f. = 1 for intercepts, and *P* < 0.05, *F*-ratio = 4.50, d.f. = 1 for slopes; abdomen: *P* < 0.0001, *F*-ratio = 102.15, d.f. = 1 for intercepts, and *P* > 0.05, *F*-ratio = 0.77, d.f. = 1 for slopes).

### Temperature and season

**Fig. 5:**
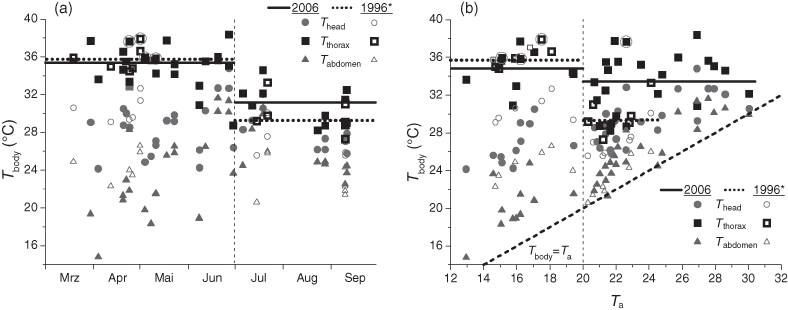
Temperature of the thorax, head and abdomen (mean of each flowering plant and day) in dependence on season (a) and ambient temperature (b) in two different years (for year 1996 see Kovac & Schmaranzer, 1996). The horizontal lines are mean thorax values of two seasons (spring and summer) or ranges of ambient temperature (*T*_a_). Values of the thorax temperature for the dandelion is marked with pink circles. Mean values between seasons (a) and between ranges of *T*_a_ (b) are significantly different (*P* < 0.05, see Results).

In Fig. 5, the mean temperatures of the three body parts during foraging in the shade are plotted against the date of observation (a) and ambient temperature (b) for each flowering plant. The *T*_th_ revealed a clear dependence on the season. The average value in the spring (March–June) as calculated from the means per stay was 35.2 ± 2.3°C, (*N* = 218). In the summer (July–September), it was only 31.4 ± 2.4°C (*N* = 127) in spite of the higher *T*_a_ in the summer. The difference could be statistically confirmed (Mann–Whitney/Wilcoxon's test, *P* < 0.0001; W = 3259.0). Testing the effect of season on average *T*_th_ per foraging stay and bee with anova (removing the effect of *T*_a_ and radiation) showed the same result (main factor season: *P* ≪ 0.0001, *F*-ratio = 247.07, d.f. = 1; covariate *T*_a_: *P* ≪ 0.0001, *F*-ratio = 40.62, d.f. = 1; covariate radiation: *P* = 0.4224, *F*-ratio = 0.65, d.f. = 1; *N* = 345). *F*-ratios indicate that season had the greatest effect followed by *T*_a_, and radiation had no effect. Plotting the average values of *T*_th_ against the ambient temperature (Fig. 5b) and calculating means for ranges of *T*_a_ according to Kovac and Schmaranzer (1996) for 12–20 °C and 20–30 °C revealed only a weak statistical difference [*T*_a_ = 12–20°C: *T*_th_ = 34.4 ± 2.3°C, (*N* = 86); *T*_a_ = 20–30°C: *T*_th_ = 33.6 ± 3.1°C, (*N* = 259); Mann–Whitney/Wilcoxon's test, *P* < 0.04; W = 9492.0].

Testing the difference between the spring and summer with all values (means per stay and bee, including values in sun and shade) confirmed the seasonal effect [spring: *T*_th_ = 35.4 ± 3.0°C, *N* = 880; summer: *T*_th_ = 32.7 ± 2.9°C, (*N* = 786); Mann–Whitney/Wilcoxon's test, *P* < 0.0001; W = 172258.0; anova, main factor season: *P* ≪ 0.0001, *F*-ratio = 508.17, d.f. = 1; covariate *T*_a_: *P* ≪ 0.0001, *F*-ratio = 378.86, d.f. = 1; covariate radiation: *P* ≪ 0.0001, *F*-ratio = 220.27, d.f. = 1; *N* = 1666].

### Type of flower

Table 1 gives an overview of body temperature and environmental parameters for each measuring day and plant divided in three classes of solar radiation (<200 W m^−2^, 200–500 W m^−2^, >500 W m^−2^). It is of special interest that bees measured on the same day in the same environment and similar *T*_a_ at different plants could exhibit remarkable differences in their thorax temperature. For example, bees foraging in the shade at apricot blossoms (*Prunus armenica* L.) had an average thorax temperature of 36.6 ± 1.8°C (*N* = 112, *T*_a_ = 18.2°C, radiation = 177 W m^−2^), whereas the thorax temperature of bees foraging on lady's smock (*Cardamine pratensis* L.) was only 34.7 ± 2.9°C (*N* = 59, *T*_a_ = 18.4°C, radiation = 186 W m^−2^; Mann–Whitney/Wilcoxon's test, *P* < 0.0001; W = 4580.0). Water-collecting bees a few metres away from the nectar foragers had the highest thorax temperatures (*T*_th_ = 37.1 ± 2.3°C, *N* = 200, *T*_a_ = 18.0°C, radiation = 151 W m^−2^; water versus *Cardamine*: Mann–Whitney/Wilcoxon's test, *P* < 0.0001; W = 3329.5; water versus *Prunus*: Mann–Whitney/Wilcoxon's test, not significant; W = 9924.5. The thorax temperature of water-collecting bees was most of the time higher than that of nectar-foraging bees on the same day and place. Another example of strongly differing thoracic temperatures were found in bees foraging in the sun on *Ranunculus* (*T*_th_ = 37.2 ± 1.3°C, *N* = 84, *T*_a_ = 23.6°C, radiation = 966 W m^−2^) and on *Crepis* (*T*_th_ = 33.2 ± 1.1°C, *N* = 58, *T*_a_ = 23.2°C, radiation = 722 W m^−2^; Mann–Whitney/Wilcoxon's test, *P* < 0.0001; W = 77.5; for further examples see Table 1).

## Discussion

### Ambient temperature and radiation

For a comprehensive description of an insect's thermoregulatory performance, it is of great advantage to investigate the entire range of ambient temperature to which it is likely to be exposed in its natural environment. Infrared thermography enabled us to measure the temperature of all three body parts of undisturbed foragers and revealed new knowledge about their thermoregulatory behaviour. An interesting result was that the bees regulated the *T*_th_ at a rather constant level in a broad range of *T*_a_ (10–27 °C) on average but showed a strong variation at a certain *T*_a_, depending on the plants from which they were foraging (Fig. 3). To our knowledge, there are only two similar investigations on this topic (Heinrich, 1979a; Kovac & Schmaranzer, 1996). Heinrich's study (1979a) is a pioneer work in this field. He measured *A. mellifera mellifera* foraging from *Eucalyptus* sp. and *A. m. adansonii* foraging from *B. pilosa* and *Petraea volubilis* (in the shade). The bees exhibited a thoracic (core) temperature of ∼30.5–33 °C at ambient temperatures of 11–22 °C. The (surface) *T*_th_ of our foragers measured in the shade was clearly higher (Fig. 3), with average values ranging from ∼35 to 33 °C. Kovac and Schmaranzer (1996) reported even higher mean thorax surface temperatures of 35–38 °C in bees foraging nectar from several plants. Water foragers measured in the same environment (Kovac *et al*., 2010) regulated the thorax to another 2–3 °C higher (*T*_a_ = 10–27°C; Fig. 3). At a *T*_a_ above 27 °C, the *T*_th_ increased somewhat more steeply in the nectar foragers than in the water foragers. At these high ambient temperatures, the bees' main problem seems not to be that their body temperature is too low. Rather, the dissipation of excessive heat becomes more important. More bees returning to the hive were shown to carry a fluid droplet at these temperatures (Cooper *et al*., 1985). Such droplets have a considerable cooling effect not only on the head but also on the thorax (Heinrich, 1979a,b). We suggest that cooling was more difficult for the nectar than for the water foragers because their head temperature became higher at a *T*_a_ above ∼27 °C (anova, *P* < 0.0001; *F*-ratio = 296.07; d.f. = 3; Fig. 3).

At very low *T*_a_, by contrast, it seems to be more important to keep the head warm. The haemolymph circulation from the warm thorax (Heinrich, 1979b, 1980a; Coelho, 1991a,b) provided the head with enough heat to prevent the *T*_hd_ from falling below ∼20 °C, which seems to be necessary for the proper functioning of physiological and neural processes. Regulation of the *T*_th_ at a high level even at low *T*_a_ allows the bees to keep the *T*_hd_ at a level high enough to guarantee a high suction speed at unlimited sources (Kovac *et al*., 2010). In nectar foragers a high nectar suction speed is generally not as important because the nectar is not available in an unlimited amount. Nectar foragers usually get only small portions of nectar per blossom and then have to fly or walk to the next blossom.

The temperature of the nectar foragers' abdomen was mostly below that of water foragers, probably because of the lower thorax temperature and perhaps because water foragers foraged much closer to the nest in the present study (Fig. 3). Heinrich (1980b, 1993) suggested that bees use a series of aortic loops in the petiole as a counter-current heat exchanger to prevent heat leakage to the abdomen in the cold. We agree with this opinion. However, the amount of heat reaching the abdomen may differ considerably. In contrast to the present study, where the abdominal temperature was not much elevated above the ambient temperature, it was considerably increased at low ambient temperatures in other previous investigations (Kovac & Schmaranzer, 1996; Kovac *et al*., 2010).

Digby (1955) investigated the factors affecting the temperature excess of dead or anaesthetised insects in artificial sunlight under laboratory conditions and found the temperature excess to vary directly with the radiation strength. This applies to living insects only in the ectothermic state. Foraging honeybees, however, are always endothermic at medium to low *T*_a_ (Figs 3; Heinrich, 1979a; Schmaranzer & Stabentheiner, 1988; Waddington, 1990; Kovac & Schmaranzer, 1996; Kovac *et al*., 2010). On average the thorax temperature excess was higher in our measurements on *A. mellifera carnica* than in an investigation on *A. m. mellifera* and *A. m. adansonii* by Heinrich (1979a). An important result of our investigation was that the bees used the heat gain from the sun to enhance their body temperature. This enables a quicker exploitation of the flowers because a high body temperature not only increases suction speed (Kovac *et al*., 2010) but also increases the bee's agility (Crailsheim *et al*., 1999; Stabentheiner & Crailsheim, 1999; Stabentheiner *et al*., 2003b) and flight muscle power output (Coelho, 1991a). However, at a high *T*_a_ of ∼30 °C our bees probably were only weakly endothermic. The thorax temperature excess in sunshine of ∼8 °C above ambient air was only ∼1.5 °C higher than the abdominal excess. The finding that in shade the thorax temperature excess was only ∼3.5 °C confirms that they were only weekly endothermic. At these high ambient temperatures the bees foraging in the sunshine are able to reach the optimal upper level of *T*_th_ for force production and takeoff of 38–39 °C (Coelho, 1991a) without much endothermic effort.

We often observed that bees preferred flowers in sunshine to flowers in shade. Our measurements of the blossom surface temperature (Fig. 4) showed that the solar radiation elevated their temperature by about 4 °C above the ambient air. Dyer *et al*. (2006) found that floral temperature can serve as an additional reward for pollinator insects when nutritional rewards are also available. However, we cannot exclude from our results that bees preferred the warmer flowers in sunshine owing to greater amounts of nectar secretion, because the production and concentration of nectar depends on ambient temperature and relative humidity (e.g. Beutler, 1953; Shuel, 1970; Núñez, 1977; Corbet *et al*., 1979a,b, 1993; Szabo, 1984; Corbet, 2003).

### Seasonal variability and type of plant

A great part of collected nectar and pollen is used to provide for the brood and young bees of the colonies. Brood rearing and colony development proceed in a special periodicity. In Central Europe the majority of the brood is reared in the spring until the beginning of the summer (e.g. Seeley, 1985; Wille, 1985; Winston, 1987; Liebig, 1994; Imdorf *et al*., 1996). During this time, colonies need huge amounts of nectar and pollen. The presence of a brood stimulates the foraging behaviour of the bees (Pankiw *et al*., 2004). We had presumed that bees foraging in the spring are better motivated and should therefore have a higher *T*_th_ (Dyer & Seeley, 1987; Stabentheiner & Schmaranzer, 1987; Schmaranzer & Stabentheiner, 1988). In Fig. 5a the mean *T*_th_ of each investigated plant is plotted against the date of measurement. The average *T*_th_ in the first period from March to June (*T*_th_ = 35.2°C) was significantly higher than that in the second period from July to September (*T*_th_ = 31.4°C). Results of Kovac and Schmaranzer (1996) lead to the same conclusion (see Fig. 5a). A similar relation between *T*_th_ and season was also found in dancing nectar and pollen foragers after their return to the hive (Stabentheiner, 2001). Plotting the *T*_th_ (average of investigated flowers on a day) in dependence on ambient temperature and dividing the *T*_a_ range into two classes of 12–20 and 20–30 °C did not show the great difference as reported by Kovac and Schmaranzer (1996; see Fig. 5b). This suggests season to be more important than ambient temperature for the observed high *T*_th_ in spring, which was confirmed by anova.

A further important result of the present study was to show the great variability in *T*_th_ on different plants that cannot be explained by differences in *T*_a_ and radiation (Table 1). An impressive example is bees foraging on crowfoot (*Ranunculus*) and hawksbeard (*Crepis*) at the same time in sunshine. Their *T*_th_ differed by 4 °C (Table 1). Another example is bees foraging from apricot blossoms (*P. armenica*) or lady's smock (*C. pradensis*) in the shade. They displayed a difference of 1.9 °C. This can only be explained by assuming different motivational states of the foragers which is related with the profitability of the source. In addition, the present results demonstrate that the body temperature at a certain plant may differ considerably at different dates. In dandelion, for example, average values per day were 37.7, 36.0, and 35.9 °C (Fig. 5; Table 1). This may have been caused by differences in nectar production as well as by differences in foraging motivation.

The thorax temperature and energy expenditure of sucrose-foraging honeybees varies markedly in direct response to the richness of food rewards and their distance from the hive (e.g. Stabentheiner & Schmaranzer, 1986, 1987, 1988; Dyer & Seeley, 1987; Schmaranzer & Stabentheiner, 1988; Waddington, 1990; Stabentheiner & Hagmüller, 1991; Underwood, 1991; Balderrama *et al*., 1992; Stabentheiner *et al*., 1995; Stabentheiner, 1996, 2001; Moffatt & Núñez, 1997; Moffatt, 2000, 2001; Sadler & Nieh, 2011). The observed thorax temperatures of nectar foragers (means ∼34–36 °C) are a bit lower than those of bees foraging 0.25–0.5 m sucrose solution (means ∼35–38 °C, Schmaranzer & Stabentheiner, 1988). However, these latter bees received sufficient food in unlimited flow at an artificial feeding place. Nectar foraging at a similar molarity is probably not as attractive because the nectar amount and flow rate of blossoms depends on ambient temperature, humidity, and other parameters. Bees have to visit many blossoms in a greater area to collect the same quantity, which requires a higher total energy expenditure to fill their crop. This probably reduces their foraging motivation and, as a consequence, the thorax temperature. The observation that a reduction of the flow rate of artificial flowers reduces the foragers' energy turnover and thus their thorax temperature (Moffatt & Núñez, 1997; Moffatt, 2000, 2001) supports this suggestion. The foragers modulate their behaviour in relation to nectar source profitability: as profitability increases, the tempo of foraging and the intensity of dancing increase (Seeley *et al*., 1991). The motivation of foragers is influenced by both the reward at the source and the demand in the hive (Seeley, 1986, 1992; Seeley & Tovey, 1994; Stabentheiner, 2001). The relative importance of these two parameters in bees foraging from flowers remains to be investigated. However, predicting the profitability of flowers for the visiting bee is very difficult as Goulson (1999) stated in his review: ‘Flowers typically exhibit a patchy distribution at a number of levels; flowers are often clustered into inflorescences, several flowers or inflorescences may be clustered on each plant, and the plants themselves are likely to be patchily distributed. Superimposed on this distribution, rewards per flower vary greatly between plants of a single species and between flowers on a single plant owing to genetic and environmental influences on reward production rates and also in response to the pattern of depletion of rewards by foragers' (for detailed literature see Goulson, 1999). In addition, the demand for nectar and pollen in a colony and between colonies may change in time and differ considerably.

However, any motivation effect on body temperature is superimposed by physiological constraints. Although bees were observed to heat their thorax up to 48 °C (Stabentheiner *et al*., 2002, 2007) they usually exhibit a *T*_th_ below 44 °C (Stabentheiner & Schmaranzer, 1987; Schmaranzer & Stabentheiner, 1988; Kovac & Schmaranzer, 1996; Kovac *et al*., 2010; Fig. 3). Lower limits for takeoff and flight in this investigation were ∼27 °C, which is somewhat lower as reported by Esch (1976) and Heinrich (1993) at ∼30 °C and obviously lower as reported by Coelho (1991b) at ∼35 °C. Bees need to increase this minimum level as nectar load increases. Temperatures at takeoff, where sucrose- and water-foraging bees are heavily loaded, are usually higher than temperatures upon landing (Schmaranzer & Stabentheiner, 1988; Kovac *et al*., 2010).

The finding that bees that were investigated on the same day in the same location sometimes displayed very different *T*_th_'s at different plants (Table 1) supports the motivation hypothesis. However, as previously mentioned, the higher spring thorax temperatures may have been caused by a greater motivation as a result of both a higher reward and a higher demand in the colony.

### Conclusion

Honeybees are always endothermic during foraging on flowers. However, at higher ambient temperatures (∼30 °C) the thoracic temperature excess is reduced to a low level and the prevention of overheating becomes more important. On average, the thorax temperature is kept rather constant at a high level in a broad range of *T*_a_ (10–27 °C) but shows a strong variation at a certain *T*_a_, depending on the plants they are foraging from. The heat gain from solar radiation is used to elevate the thorax temperature during foraging and, in this way, probably improves the agility and speed of food exploitation. A high thorax temperature enables elevation of the head temperature and keeps the abdomen temperature some degrees above the ambient air. This improves physiological processes involved in food uptake, respiration, and energy supply. We suggest that the higher thorax temperature in spring is mainly caused by a higher foraging motivation as a result of the higher demand for nectar and pollen in the colony.
